# A Rare Case of Cavitary Lesion of the Lung Caused by* Mycoplasma pneumoniae* in an Immunocompetent Patient

**DOI:** 10.1155/2017/9602432

**Published:** 2017-08-21

**Authors:** Muhammad Kashif, Rizwan Ahmed Dudekula, Misbahuddin Khaja

**Affiliations:** ^1^Division of Pulmonary and Critical Care Medicine, Department of Medicine, Bronx Lebanon Hospital Center Affiliated with Icahn School of Medicine at Mount Sinai, Bronx, NY, USA; ^2^Department of Medicine, Bronx Lebanon Hospital Center Affiliated with Icahn School of Medicine at Mount Sinai, Bronx, NY, USA

## Abstract

*Mycoplasma pneumoniae* is an atypical bacterium that most commonly causes upper respiratory tract infections, but it can also cause pneumonia, referred to as “walking pneumonia.” Although cavitary lesions are present in a wide variety of infectious and noninfectious processes, those attributable to* M*.* pneumoniae* are extremely uncommon; thus, to date, epidemiological studies are lacking. Here, we present a rare case of a 20-year-old male, referred to us from a psychiatric facility for evaluation of a cough, who was found to have a cavitary lesion in the right upper lobe. An extensive workup for cavitary lesion was negative, but his mycoplasma IgM level was high. A computed tomography (CT) of the chest confirmed the presence of a cavitary lesion. After treatment with levofloxacin antibiotics, a follow-up CT showed complete resolution of the lesion. Our case is a rare presentation of mycoplasma pneumonia as a cavitary lesion in a patient without any known risk factors predisposing to mycoplasma infection. Early recognition and treatment with an appropriate antibiotic may lead to complete resolution of the cavitary lesion.

## 1. Introduction

*Mycoplasma pneumoniae* infection is mild and self-limiting. Although the term “mycoplasma” is widely used to refer to any organism within the class Mollicutes, only four species—*Mycoplasma pneumoniae*,* Mycoplasma hominis*,* Mycoplasma genitalium*, and* Ureaplasma urealyticum*—are well-established human pathogens [1].

*M*.* pneumoniae* is one of the most common causes of atypical pneumonia, which accounts for up to 35% of pneumonia cases in the United States and is responsible for up to 18% of cases in which patients need to be hospitalized.* M*.* pneumoniae* is transmitted from person to person by infected respiratory droplets during close contact. The incubation period after exposure averages 2 to 3 weeks [2].

Both noninfectious and infectious conditions can cause cavitary lesions in the lung. Such lesions are a rare complication of* M*.* pneumoniae*. Primarily severe* M*.* pneumoniae* infection is associated with predisposing factors, such as a compromised immune system, chronic steroid use, chemotherapy, or active smokers with lung disease [3].

## 2. Case Presentation

A 24-year-old male presented to our emergency department with worsening cough, fever, and chills of one-day duration. The patient reported cough for one week that progressively worsened, with mucoid phlegm associated with low-grade fever, chills, and shortness of breath of 1-day duration. He denied hemoptysis, gastrointestinal symptoms, and chest pain and reported no bird exposure, skin rash, arthralgia, recent travel, or sick contacts. His medical history was significant for mild persistent asthma and schizophrenia. He had no prior surgeries and had resided in a psychiatric facility for 2 years with no occupational exposure to chemicals or toxins. He was a former polysubstance abuser and was in abstinence for 2 years. He denied smoking cigarettes, using illicit drugs, or abusing alcohol and had no reported allergies. His medications included divalproex sodium, clozapine, zolpidem, albuterol, and fluticasone aerosol inhaler. He had a negative tuberculin skin test 6 months prior to admission for the current complaint.

**Figure 1:**
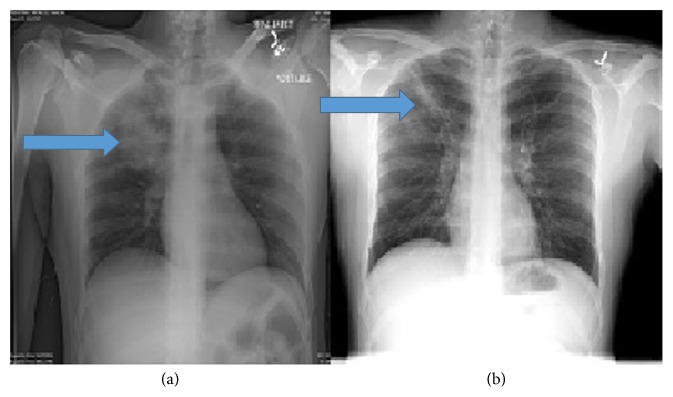
(a) Chest X-ray performed on admission, showing right upper lobe consolidation. (b) Chest X-ray performed at 2-month follow-up, showing right upper lobe scarring and improvement in consolidation.

**Figure 2:**
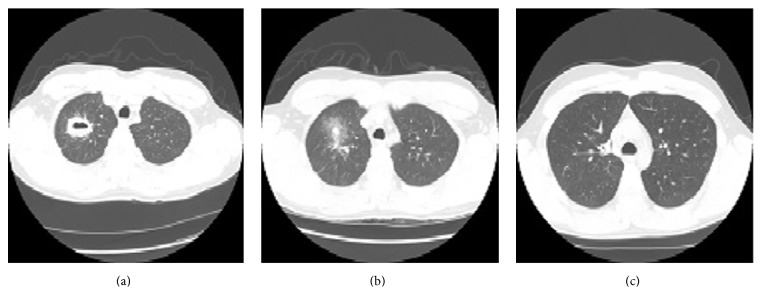
(a) Chest axial view of CT performed on the day of admission, showing a thick-wall cavitary lesion (6.0 × 1.6 × 3.5 cm) in the right upper lobe of the lung. (b) Chest axial view of CT performed at 2-month follow-up, showing foci with ground glass opacity in the anterior right upper lobe of the lung surrounding a 9.9 mm nodular density. (c) Chest axial view of a CT performed at 3-month follow-up, showing complete resolution of the right upper lobe cavitary lesion.

A physical examination revealed a young man of average built, with a temperature of 100.4 F, pulse rate of 78/min, respiratory rate of 20/min, and blood pressure of 110/66 mmHg; he also showed 96% saturation on ambient air. He appeared lethargic, with no conjunctival pallor, cyanosis, nuchal rigidity, skin eruptions, or palpable lymphadenopathy. Bilateral air entry was evident on auscultation of lungs, with fine rales on the right side. A precordial examination revealed normal heart sounds, with no murmur, rub, or gallop. An abdominal exam revealed no organomegaly, and a neurological examination showed no motor or sensory neurological deficits. An initial laboratory examination showed neutrophilic leukocytosis (white blood cell count, 18,000/mm^3^ with 74% neutrophils) and elevated blood urea nitrogen (25 mg/dL) with lactic acidosis (2.5 mmol/L). He had no anemia (hemoglobin, 13.5 g/dL), thrombocytopenia (157,000/*μ*L), or renal failure (creatinine, 0.6 mg/dL). A urine toxicology screen was negative for any drugs. His initial chest radiograph showed right upper lobe consolidation (Figure 1(a)). He was admitted to the hospital for pneumonia, which was managed with vancomycin, piperacillin-tazobactam, and azithromycin. Further evaluation by chest computed tomography (CT) showed a thick-walled, right upper lobe cavitary lesion (Figure 2(a)). Fiberoptic bronchoscopy (FOB) with bronchoalveolar lavage (BAL) was performed to test for associated infections or noninfectious processes. A transbronchial biopsy of the lung showed diffuse lymphocytic infiltrates. Cultures for common bacteria,* Mycobacterium tuberculosis*, and fungi were negative, as were cultures for respiratory syncytial virus, influenza viruses, parainfluenza viruses, adenoviruses, and vasculitis workup as shown in Table 1. Serology performed to detect other infections indicated a cold agglutinin titer of 1 : 320 and an* M*.* pneumoniae* IgM antibody titer of 1 : 1,280. At this point, antibiotic treatment with oral azithromycin was continued and the remaining antibiotics were discontinued. The patient's condition did not improve clinically, so levofloxacin was started and the patient was discharged to the psychiatric facility on oral medication for 2 weeks. Follow-up chest radiograph two months later showed right upper lobe scarring and improvement in consolidation (Figure 1(b)). Follow-up CT scan two months later showed foci with ground glass opacity in the anterior right upper lobe of the lung surrounding a 9.9 mm nodular density and three months later showed complete resolution of the cavitary lesion without sequelae (Figures 2(b) and 2(c)); the antibody titer for* M*.* pneumoniae* IgM was also reduced (1 : 160) 3 months later.

## 3. Discussion

*M*.* pneumoniae* is one of the most common causes of lower respiratory tract infections, accounting for up to 40% of respiratory tract infections in the community [4]. Those most at risk include children younger than age 5 and older adults with diseases that compromise their immune systems, such as HIV; are on chronic steroids, immunotherapy, or chemotherapy; have sickle cell disease; are smokers; or have lung disease. Our patient did not have any of these risk factors, increasing the rarity of this case [3].

*M*.* pneumoniae* infection may present with a variety of symptoms, including headache, nausea, vomiting, diarrhea, fever, articular pains, myalgia, ear pain, sore throat, and cough. Extra pulmonary abnormalities seen in mycoplasma infections include hemolysis, skin rash, chemical hepatitis, encephalitis, meningitis, rhythm disturbance, congestive heart failure, myocarditis, glomerulonephritis, polyarthralgia, myalgia, otitis media, and bullous myringitis [5, 6]. The fatal complications of* M*.* pneumoniae* infection are not well established but include acute respiratory distress syndrome, disseminated intravascular coagulation, hemophagocytic syndrome, acute disseminated encephalomyelitis, and Stevens-Johnson syndrome [7, 8].

Cavitary lesions associated with specific diseases are frequently described as being “thick walled” or “thin walled” and of noninfectious or infectious origin. Some noninfectious causes are squamous cell carcinoma of lung, lymphoma, Kaposi sarcoma, and metastatic disease. Other noninfectious conditions include Wegener's granulomatosis, sarcoidosis, and Langerhans cell histiocytosis. Infectious causes of cavitary lesions include necrotizing pneumonia, lung abscess caused by* Streptococcus pneumoniae*,* Staphylococcus aureus*,* Haemophilus influenzae*,* Klebsiella pneumoniae*, and septic pulmonary emboli. Uncommon bacterial infections that can cause cavitary lesions are* Actinomyces*,* Nocardia*,* Burkholderia pseudomallei*,* Rhodococcus equi*, and nontuberculous and tuberculous mycobacteria. Fungal infections such as* Candida*,* Aspergillus* species,* Mucor*,* Histoplasma capsulatum*,* Blastomyces dermatitidis*,* Coccidioides*,* Paracoccidioides brasiliensis*,* Cryptococcus neoformans*, and* Pneumocystis jiroveci* may also cause cavitary lesions [9, 10].

In our case, there was no renal involvement, and a lung biopsy revealed no tumors or granulomas. These results effectively exclude Wegener's granulomatosis, sarcoidosis, and malignancy. All of our patient's bacterial, viral, and fungal cultures were negative, excluding most infectious etiologies. Cultures were negative for typical and atypical mycobacteria, but the patient had elevated cold agglutinin and* M*.* pneumoniae* antibody titers. The formation of cold agglutinins is a nonspecific early IgM reaction against the erythrocyte I antigen, where a titer ≥ 1 : 64 represents a positive test [11].

There were no distinguishing clinical or radiographic manifestations that confidently diagnose* M*.* pneumoniae* versus other causes of atypical pneumonia, such as* Chlamydia* or* Legionella* species. Because they lack a cell wall, mycoplasmas are not visible on Gram staining. Instead,* M*.* pneumoniae* infection is diagnosed by serological antibody tests, including a complement fixation test, passive agglutination test, and indirect agglutinin test, either alone or in combination [12]. The gold standard for serologic diagnosis is a fourfold change in antibody titers over time (IgM antibodies rise earlier than IgG antibodies). Direct polymerase chain reaction (PCR) to detect genomic DNA may be highly sensitive and specific for* M*.* pneumoniae* in patients with respiratory tract infections [13].

The therapeutic mainstays for suspected* M*.* pneumoniae* infection are macrolide antibiotics, doxycycline, or a fluoroquinolone [14, 15]. Our patient was given azithromycin for 5 days, but his symptoms did not improve. He was then given a longer course of levofloxacin for 14 days, and clinical and radiologic findings subsequently improved. Antibiotic resistance although uncommon may be suspected in cases of unresponsiveness to macrolides [16].

## 4. Conclusions

*M*.* pneumoniae* is a well-recognized cause of pneumonia, but it is rarely associated with cavitary lesions, which are extremely rare in immune competent patients. We report a rare case of cavitary pneumonia caused by mycoplasma. Early identification and treatment led to complete resolution of the cavitary lesion.

## Figures and Tables

**Table 1:** Pertinent laboratory findings.

Serum anti-RSV IgM	0.64
Serum anti-RSV IgG	1.4
Nasal influenza A/B swab	Negative
Anti-ribosomal P antibody	Negative
Anti-nuclear antibody	Negative
Anti-DNA antibody	Negative
Smooth muscle antibody	Negative
Anti-scleroderma 70 antibody	Negative
Myeloperoxidase	Negative
Proteinase 3 antibody	Negative
Cold agglutinin antibody	Negative
Rheumatoid factor	Negative
Anti-cyclic citrullinated antibody	Negative
HIV	Negative
*Chlamydiapneumoniae* IgM	<1 : 20
Chlamydial pneumonia IgG	<1 : 64
*Legionella* antigen, urine	Negative
*Histoplasma* antigen, urine	Negative
Blood, urine, and respiratory culture	Negative
BAL cultures and acid fast bacilli stains	Negative
Cryoglobulin level	Negative
*M*. *pneumoniae* IgM antibody titer	1 : 1,280
Cold agglutinin titer	1 : 320

## Abbreviations

CT: Computed tomography
*M*.* pneumoniae*: * Mycoplasma pneumoniae*.
